# Improvement in Non‐Traumatic, Out‐Of‐Hospital Cardiac Arrest Survival in Detroit From 2014 to 2016

**DOI:** 10.1161/JAHA.118.009831

**Published:** 2018-08-21

**Authors:** Spencer May, Liying Zhang, Dan Foley, Erin Brennan, Brian O'Neil, Ethan Bork, Phillip Levy, Robert Dunne

**Affiliations:** ^1^ Department of Emergency Medicine Wayne State University School of Medicine Detroit MI; ^2^ Department of Family Medicine and Public Health Sciences Wayne State University School of Medicine Detroit MI

**Keywords:** African American, Black, resource‐limited, cardiac arrest, emergency medical services, out‐of‐hospital cardiac arrest, sudden cardiac arrest, surveillance, survival, survival rate, urban, Cardiopulmonary Resuscitation and Emergency Cardiac Care, Risk Factors, Quality and Outcomes, Health Services, Mortality/Survival

## Abstract

**Background:**

In 2002, the out‐of‐hospital cardiac arrest (OHCA) survival rate in Detroit was the lowest in the nation. Concerted efforts sought to improve the city's chain of survival with a focus on emergency medical services (EMS). This study assesses the impact on OHCA survival rates and describe factors associated with survival.

**Methods and Results:**

Data for non‐traumatic OHCA cases in Detroit from 2014 to 2016 were extracted from CARES (Cardiac Arrest Registry to Enhance Survival). Chi‐squared tests, non‐parametric tests, and a multivariable logistic regression analysis were employed to examine the associations between overall survival and its covariates. A total of 2359 non‐traumatic OHCA cases were examined. The overall survival rate increased from 3.7% in 2014 to 5.4% in 2015, and 6.4% in 2016 (*P*<0.01), reflecting a 73% improvement in survival over the 3‐year period. EMS median on‐scene time decreased over the study period, while the rate at which EMS initiated cardiopulmonary resuscitation and applied an automated external defibrillator (AED) greatly increased (*P*<0.001). The factors significantly associated with survival were female sex (odds ratio=1.70, *P*<0.05), a public setting (odds ratio=2.31, *P*<0.01), an EMS witness (odds ratio=6.18, *P*<0.01), and the presence of an initial shockable rhythm (odds ratio=1.88, *P*<0.05).

**Conclusions:**

From 2014 to 2016, the overall survival rate for OHCA patients in Detroit, MI significantly improved. Our results suggest that an improved chain of survival may explain this progress. This study is an example of how OHCA data analysis and EMS improvement can improve end OHCA outcomes in a resource‐limited urban setting.

**Clinical Perspective**

**What Is New?**

- In 2002, the out‐of‐hospital cardiac arrest survival rate in Detroit was the lowest in the nation.
- Since 2013, CARES (Cardiac Arrest Registry to Enhance Survival) has tracked out‐of‐hospital cardiac arrest outcomes in Detroit.
- From 2014 to 2016, out‐of‐hospital cardiac arrest survival in Detroit increased dramatically after CARES‐informed changes were made to the city's emergency medical services.

**What Are the Clinical Implications?**

- This study suggests that coordinated and data‐driven emergency medical service improvements can more rapidly initiate the chain of survival and greatly improve end outcomes for out‐of‐hospital cardiac arrest patients in urban settings with limited resources.

## Introduction

Out of hospital cardiac arrest (OHCA) is a major cause of death in the United States.^1^ An estimated 395 000 cases of OHCA occur in the United States every year, and 2005 to 2010 data from the CARES (Cardiac Arrest Registry to Enhance Survival) showed that the nation's overall survival rate to hospital discharge was 9.6%.^2^ By targeting elements predictive of survival, improvements to OHCA care over the past decade has resulted in greater national overall survival rates.^3^

A 2002 study conducted in the city of Detroit reported that 6% (n=28) of patients survived to hospital admission and 0.2% (n=1) of patients survived to hospital discharge among 471 confirmed OHCA cases.^4^ This survival rate reflects the lowest ever reported among similar cities in the United States and highlights how, at the time, OHCA was an almost uniformly fatal event in Detroit, MI.

Detroit OHCA data during the next decade are limited, as many of the city's systems were less functional during the city's financial decline and ultimate bankruptcy.^5^ Since the 2013 bankruptcy, the city of Detroit has made significant improvements in emergency medical services (EMS) care for OHCA patients. One of the key drivers was tracking OHCA care and outcomes data in CARES and partnering with the Save‐MI‐Heart collaborative. In 2013 Save‐MI‐Heart,^6^ a state‐based partnership dedicated to improving cardiac arrest survival, provided support for rigorous data collection of Detroit OHCA cases, which was then used to prompt changes in cardiac arrest care based on successes from other CARES sites. New city leadership was also data focused and committed to improving patient care.

Changes to the emergency response system were implemented concurrently from 2014 through 2016. Among them, an emergency medical dispatch priority system was established for the city of Detroit in 2015 that included standardized, comprehensive pre‐arrival instructions (the city currently uses medical dispatch priority system Pro‐QA 13.4) and training of all 911 operators. This new system replaced an older system where call‐takers had no medical training and is more comprehensive than what is required by Michigan statute.

For the first time in the history of the city, non‐transporting fire units were used in a medical role, with dispatch to all high priority (echo and delta level) medical dispatch priority system calls. From February 2015 to April 2017, over 800 single‐role fire fighters were trained and licensed as medical first responders (MFRs). Private industry facilitated the donation of ambulances to the city and a formal agreement was made with private EMS providers to support the city's EMS system during peak hours.^7^ The city also committed to a process improvement program and partnered with Wayne State University School of Medicine's Department of Emergency Medicine to support an EMS fellowship.

The current project aims to examine the changes of OHCA survival rates in Detroit from 2014 to 2016, using CARES data. The factors associated with survival in Detroit during this study period are also explored. Because of its historically low OHCA survival, Detroit makes an ideal case study to evaluate what may work at a system level to improve outcomes.

## Methods

Data used in the current study are derived from CARES website databases.^8^ CARES protocols govern access to this study's data set; requests from qualified researchers trained in human subject confidentiality may be sent to Kimberly Vellano of the CARES Program at khauste@emory.edu.^9^ Requests for access to the study's analytical methods can be directed to the corresponding author. The use of CARES in Detroit was reviewed by the Wayne State University Institutional Review Board (IRB) as exempt.

### Setting

Detroit is the largest city on the US‐Canada border. In 2016, the estimated population in the city of Detroit was ≈672 795, with 13% of the population aged ≥65 years.^10^ The owner‐occupied housing unit rate was 48.2% housing units with a median household income of ≈$26 249, with 39.4% of residents living below the poverty level, and with 84% of the population identifying as African‐American.^10^, ^11^

The Detroit Fire Department operates 6 non‐transporting squads and 27 fire engines all licensed at the MFR level. There are 27 Basic Life Support (BLS) ambulances and 9 Advanced Life Support (ALS) ambulances operated by the fire department. In addition, there are 8 ambulances provided by 4 private companies that provide peak‐hour coverage and are dispatched by the city. Each call for service that is prioritized at the echo or delta level has the nearest ambulance and MFR apparatus dispatched. There is no preferential dispatch of ALS units because of the small number available and the size of the response area.

Cardiac arrest care is performed under standard protocols and all cases where resuscitation is attempted are included in the Detroit CARES data, including those that were terminated in the field. There are standard dead‐on‐scene protocols when no resuscitation will be attempted. There are ALS and BLS protocols for cardiac arrest with BLS units performing 3 initial cardiopulmonary resuscitation (CPR) cycles (6 minutes) on scene before preparing for transport.^12^ The city uses the SafetyPad prehospital electronic health record system.

### Data Extraction and Quality Assessment

To measure the outcomes and progress of patients who experience OHCA, the Center for Disease Control and Prevention collaborated with the Emory University School of Medicine in 2004 to develop a national registry of OHCA data: CARES.^2^ CARES adopts Utstein‐style reporting guidelines, which provide a standard, structured framework to collect and report data of patents with cardiac arrest.^13^, ^14^ CARES is one of the largest OHCA registries and quality improvement programs in the world, with >1800 hospitals and 1400 EMS agencies from 23 participating states.^8^

Non‐traumatic OHCA cases in the city of Detroit from January 1, 2014 to December 31, 2016 were extracted from the CARES database, with etiologies of arrest that are categorized as presumed cardiac cause, drowning or submersion, drug overdose, electrocution, exsanguination or hemorrhage, and respiratory or asphyxia. We excluded all cases where EMS terminated their efforts according to do‐not‐resuscitate orders. Both pediatric patients and adult patients were included. According to these criteria, a total of 2359 cases were identified and analyzed.

Data validation for these records is performed by both automated checks in the CARES system as well as an annual review of all CARES data for missing items, outliers, and conflicting values. An agreement between Detroit EMS and Wayne State University School of Medicine's Department of Emergency Medicine enabled the department to provide its expertise to EMS data management, including personnel for auditing the uploaded prehospital data in CARES and coordinating the hospital outcome data.

### Statistical Analysis

First, descriptive statistics were calculated for variables of interest. Statistical significances of differences were tested using ANOVA or t test for continuous variables and chi‐square test for categorical variables. We categorized age into 5 groups according to 5th, 25th, 50th, and 75th percentiles (ie, <20, 21–50, 51–62, 63–74, >75 years) for the purpose of analysis. To examine the significance of differences in survival percentage point increase over the 3‐year study period, *P*‐value was calculated based on chi‐square test of 3 proportions of survival. For the time variables (eg, response time, on‐scene time, and transport time), the medians and interquartile ranges (IQR) are presented. Response time is defined as the difference between the time of dispatch and the time of arrival on‐scene; on‐scene time is defined as the difference between the time of arrival on‐scene and the time of leaving the scene; and transport time is defined as the difference between the time of leaving the scene and the time of arrival at the hospital. The significance of differences in response, on‐scene, and transport times over years was tested using non‐parametric testing.

Second, a multivariable logistic regression analysis was performed to examine the association between factors and survival as the dependent variable. The relevant variables with *P*‐values <0.05 in the bivariate analysis (eg, year, age group, sex, location of arrest, arrest being witnessed, and initial shockable rhythm) were included in multivariable logistic regression models. The overall survival rate is defined as the number of patients surviving to hospital discharge divided by the total number of OHCA patients during the study period. Adjusted odds ratio (OR) and their 95% confidence interval (95% CI) were also calculated. The Stukel test for goodness‐of‐fit was implemented to examine the model fit.^15^, ^16^ All statistical analyses were performed using SAS version 9.4 (SAS Institute, Inc, Cary, NC).

## Results

The Figure presents the overall survival rates for OHCA in the city of Detroit, demonstrating a 73% increase over the 3‐year study period (3.7% in 2014, 5.4% in 2015, and 6.4% in 2016; *P*=0.004). For context, national rates are also included, showing better but static OHCA survival rates over the same time frame (10.8% in 2014 and 10.5% in 2015 and 2016).^8^

**Figure 1:**
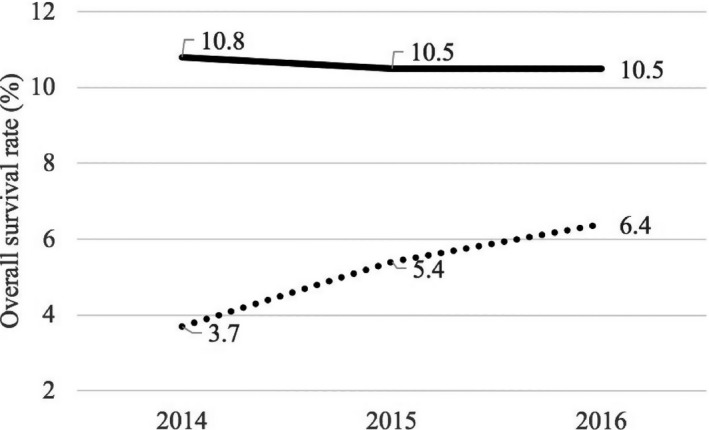
Comparison of the overall survival rates of out‐of‐hospital cardiac arrest in the city of Detroit with national rates, 2014–2016. Data derived from the Non‐traumatic OHCA Utstein Survival Report from the CARES database.^8^ 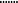, Detroit; 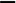, National.

Table 1 presents characteristics of the OHCA population. The mean age was 60.4 years, 54.9% were male, 62.9% were black, and 86.7% were presumed to have a cardiac cause of their arrest. From 2014 to 2016, statistically significant increases were observed in the proportion of public settings as the arrest location, and the proportion of bystander‐witnessed arrests, while a statistically significant decrease was observed in the proportion of unwitnessed arrests. EMS‐related care factors showed significant improvement. In 2014, the proportion of CPR initiation by medical first responders after arrest was 3.7%. This proportion sharply rose to 10.1% in 2015 and to 20.7% in 2016 (*P*<0.001). Other increases were observed in the proportion of arrests with an automated external defibrillator (AED) applied by a medical first responder (28.3% in 2014, 59.6% in 2015, and 83.2% in 2016, *P*<0.001), and in the proportion of responding EMS personnel who first defibrillated a patient (18.4% in 2014, 22.8% in 2015, and 29.1% in 2016, *P*<0.001). Furthermore, the proportion of arrests with an AED applied before EMS arrival increased (6.5% in 2014, 11.5% in 2015, and 21.4% in 2016, *P*<0.001).

**Table 1:** Characteristics of Out‐Of‐Hospital Cardiac Arrest Patients in Detroit From January 1, 2014 to December 31, 2016

Variables	Total n (%) Mean {±SD}	2014 n (%) Mean {±SD}	2015 n (%) Mean {±SD}	2016 n (%) Mean {±SD}	*P* Value
Total of number	2359	821	775	763	
Presumed cardiac patient					
No	314 (13.3)	79 (9.6)	111 (14.3)	124 (16.3)	<0.001^a^
Yes	2045 (86.7)	742 (90.4)	664 (85.7)	639 (83.7)	
Age, (y)	60.4 {**±**20.5}	61.1 {**±**19.9}	59.3 {**±**21.3}	60.6 {**±**20.3}	0.224
0 to 20	115 (4.9)	35 (4.3)	44 (5.7)	36 (4.7)	
21 to 50	479 (20.4)	158 (19.4)	171 (22.2)	150 (19.7)	
51 to 62	607 (25.9)	210 (25.8)	185 (24.1)	150 (19.7)	
63 to 74	582 (24.8)	213 (26.1)	181 (23.5)	188 (24.7)	
>75	561 (23.9)	199 (24.4)	188 (24.5)	174 (22.9)	
Sex					
Female	1063 (45.1)	373 (45.4)	352 (45.4)	338 (44.3)	0.876
Male	1296 (54.9)	448 (54.6)	423 (54.6)	425 (55.7)	
Black					
No	876 (37.1)	361 (44.0)	336 (43.4)	179 (23.5)	<0.001^a^
Yes	1483 (62.9)	460 (56.0)	439 (56.7)	584 (76.5)	
Location of arrest					
Home/residence	1803 (89.4)	637 (91.1)	615 (91.2)	551 (85.6)	<0.001^a^
Public setting	214 (10.6)	62 (8.9)	59 (8.8)	93 (14.4)	
Arrest witnessed					
Bystander witnessed	662 (28.1)	241 (29.4)	180 (23.2)	241 (31.6)	<0.001^a^
Witnessed by EMS	219 (9.3)	80 (9.7)	61 (7.9)	78 (10.2)	
Unwitnessed	1478 (62.7)	500 (60.9)	534 (68.9)	444 (58.2)	
Who initiated CPR					
Bystander	711 (30.1)	254 (30.9)	199 (25.7)	258 (33.8)	<0.001^a^
Medical first responder	266 (11.3)	30 (3.7)	78 (10.1)	158 (20.7)	
EMS	1382 (58.6)	537 (65.4)	498 (64.3)	347 (45.5)	
Was an AED applied before EMS arrival					
No	2054 (87.1)	768 (93.5)	686 (88.5)	600 (78.6)	<0.001^a^
Yes	305 (12.9)	53 (6.5)	89 (11.5)	163 (21.4)	
Who first applied AED (N=305)					
Medical first responder	202 (66.7)	15 (28.3)	53 (59.6)	134 (83.2)	<0.001^a^
Bystander	101 (33.3)	38 (71.7)	36 (40.4)	27 (16.8)	
Missing cases	2	0	0	2	
Who first defibrillated the patient					
Bystander	25 (1.1)	15 (1.8)	4 (0.5)	6 (0.8)	<0.001^a^
Medical first responder	59 (2.5)	4 (0.5)	16 (2.1)	39 (5.1)	
Responding EMS personnel	550 (23.3)	151 (18.4)	177 (22.8)	222 (29.1)	
Not applicable	1725	651	578	496	
Initial shockable rhythm					
No	2071 (87.8)	730 (88.9)	686 (88.5)	655 (85.8)	0.132
Yes	288 (12.2)	91 (11.1)	89 (11.5)	108 (14.2)	

AED indicates automated external defibrillator; CPR, cardiopulmonary resuscitation; EMS, emergency medical services.

a *P*<0.05.

Table 2 displays survival data for all 2359 non‐traumatic OHCA cases that occurred in the city of Detroit during the study period from January 1, 2014 to December 31, 2016. In addition to the improvements in OHCA overall survival, bystander‐witnessed survival rates showed a significant increase (5.6% in 2014, 9.1% in 2015, and 12.2% in 2016; *P*=0.004). The rate of survival to hospital admission also increased (15.3% in 2014, 19.6% in 2015, and 20.5% in 2016; *P*=0.017). Both median response time and on‐scene time declined significantly over the study period.

**Table 2:** Survival Rates of Out‐Of‐Hospital Cardiac Arrest Patients in Detroit From January 1, 2014 to December 31, 2016

Variables	Total n (%) Median [IQR]	2014 n (%) Median [IQR]	2015 n (%) Median [IQR]	2016 n (%) Median [IQR]	*P* Value
Total of number	2359	821	775	763	
Overall survival	121 (5.1)	30 (3.7)	42 (5.4)	49 (6.4)	0.004^a^
Survival to hospital admission	433 (18.4)	125 (15.3)	152 (19.6)	156 (20.5)	0.017^a^
Survival to hospital discharge	121/433 (27.9)	30/125 (24.0)	42/152 (27.6)	49/156 (31.4)	0.386
Bystander witnessed survival	79/881 (9.0)	18/321 (5.6)	22/241 (9.1)	39/319 (12.2)	0.014^a^
Unwitnessed survival	42/1478 (2.8)	12/500 (2.4)	20/534 (3.8)	10/444 (2.3)	0.288
Utstein survival^b^	20/144 (13.9)	5/40 (12.5)	3/38 (7.9)	12/66 (18.2)	0.329
Utstein bystander survival^c^	8/58 (13.8)	1/20 (5.0)	2/10 (20.0)	5/28 (17.9)	0.365
Response time (in minutes)	6.0 [4.0–8.0]	7.0 [5.0–10]	6.0 [4.0–8.0]	6.0 [4.0–8.0]	<0.001^a^
On‐scene time (in minutes)	15.0 [10.4–21.0]	17.0 [11.0–22.0]	15.0 [11.0–21.0]	13.0 [9.8–19.0]	<0.001^a^
Transport time (in minutes)	7.0 [5.0–10.0]	7.0 [5.0–10.0]	7.0 [5.0–10.0]	7.0 [4.8–9.0]	<0.001^a^

IQR indicates interquartile range.

a *P*<0.05.

b Witnessed by bystander and found in a shockable rhythm

c Witnessed by bystander, found in shockable rhythm, and received some bystander intervention (cardiopulmonary resuscitation by bystander and/or automated external defibrillators applied by bystander).

Table 3 presents the associations between the overall survival and relevant factors. The survival was significantly associated with the year, age, sex, and location, along with the arrest being witnessed, and the presence of an initial shockable rhythm. OHCA patients aged 21 to 50 years had a higher survival proportion than other age groups. For instance, the proportion of survival was 7.7% in the 21‐ to 50‐year age group while it was 2.8% in patients >75 years. (*P<*0.01). Male patients had a lower chance of survival than female patients (4.4% versus 6.2%, *P*<0.05). The majority of OHCA occurred at a home or residence (89.4%). Patients who arrested at their residence had a lower chance of survival compared with those whose OHCA occurred in a public setting (4.8% versus 11.7%, *P*<0.001). Unwitnessed arrests had a lower chance of survival than bystander‐witnessed and EMS‐witnessed arrests (2.8% versus 7.2% and 14.2%, *P*<0.001). Patients with initial shockable rhythm were more likely to survive (10.4% versus 4.4%, *P*<0.001).

**Table 3:** Association Between Survival and Correlates of Out‐Of‐Hospital Cardiac Arrest in Detroit, 2014–2016

Variables	Total, n (%)	Survival to Hospital Discharge		*P* Value
		Died, n (%)	Alive, n (%)	
Total of number (N)	2359	2238 (94.9)	121 (5.1)	
Year				
2014	821 (34.8)	791 (96.4)	30 (3.6)	0.040^a^
2015	775 (32.9)	733 (94.6)	42 (5.4)	
2016	763 (32.3)	714 (93.6)	49 (6.4)	
Age				
Mean (SD), y	60.4 (20.5)	60.5 (20.9)	54.5 (19.2)	0.008^a^
0 to 20	113 (4.8)	107 (94.7)	6 (5.3)	
21 to 50	481 (20.5)	444 (92.3)	37 (7.7)	
51 to 62	607 (25.9)	571 (94.1)	36 (5.9)	
63 to 74	582 (24.8)	556 (95.5)	26 (4.5)	
>75	561 (23.9)	545 (97.2)	16 (2.8)	
Sex				
Female	1063 (45.1)	997 (93.8)	66 (6.2)	0.031^a^
Male	1296 (54.9)	1241 (95.8)	55 (4.4)	
Black				
No	876 (37.1)	832 (95.0)	44 (5.0)	0.857
Yes	1483 (62.9)	1406 (94.8)	77 (5.2)	
Location of arrest				
Home/residence	1803 (89.4)	1716 (95.2)	87 (4.8)	<0.001^a^
Public setting	214 (10.6)	189 (88.3)	25 (11.7)	
Arrest witnessed				
Bystander witnessed	662 (28.1)	614 (92.8)	48 (7.2)	<0.001^a^
Witnessed by EMS	219 (9.3)	188 (85.8)	31 (14.2)	
Unwitnessed	1478 (62.7)	1436 (97.2)	42 (2.8)	
Who initiated CPR				
Bystander	711 (30.1)	675 (94.9)	36 (5.1)	0.708
Medical first responder	266 (11.3)	255 (95.9)	11 (4.1)	
EMS	1382 (58.6)	1308 (94.7)	74 (5.3)	
Was an AED applied before EMS arrival				
No	2054 (87.1)	1949 (94.9)	105 (5.1)	0.921
Yes	305 (12.9)	289 (94.8)	16 (5.2)	
Who first applied AED (N=305)				
Medical first responder	202 (66.7)	192 (95.1)	10 (4.9)	0.716
Bystander	101 (33.3)	95 (94.1)	6 (5.9)	
Missing cases	2	2	0	
Who first defibrillated the patient				
Bystander	25 (1.1)	21 (84.0)	4 (16.0)	0.201
Medical first responder	59 (2.5)	53 (89.8)	6 (10.2)	
Responding EMS personnel	550 (23.3)	511 (92.9)	39 (7.1)	
Not applicable	1725	1653	72	
Initial shockable rhythm				
No	2071 (87.8)	1980 (95.6)	91 (4.4)	<0.001^a^
Yes	288 (12.2)	258 (89.6)	30 (10.4)	

AED indicates automated external defibrillator; CPR, cardiopulmonary resuscitation; EMS, emergency medical services.

a *P*<0.05.

Multivariable logistic regression analysis results are presented in Table 4 and indicate that the year, patient sex, location of the arrest, arrest witness, and the presence of an initial shockable rhythm were the main factors associated with survival, with age no longer impacting outcome once these variables were accounted for. OHCA patients with an initial shockable rhythm were more likely to survive than those without (OR=1.875, *P*=0.011). The Stukel test produced a Wald chi‐square of 0.21 (degrees of freedom=2), yielding a *P*‐value of 0.90; indicating the model is a good fit.

**Table 4:** Multivariable Logistic Regression for Survival of Out‐Of‐Hospital Cardiac Arrest in Detroit, 2014–2016

Variables	OR	95% CI	*P* Value
Year			
2014			
2015	1.677	(1.003, 2.804)	0.049^a^
2016	1.665	(1.008, 2.749)	0.047^a^
Age group			
0 to 20			
21 to 50	1.218	(0.488, 3.040)	0.673
51 to 62	0.913	(0.364, 2.290)	0.846
63 to 74	0.610	(0.235, 1.582)	0.309
>75	0.414	(0.150, 1.139)	0.088
Sex			
Male			
Female	1.695	(1.126, 2.553)	0.012^a^
Location of arrest			
Home/residence			
Public setting	2.309	(1.386, 3.845)	0.001^a^
Arrest witnessed			
Unwitnessed			
Bystander witnessed	2.750	(1.721, 4.393)	<0.001^a^
Witnessed by EMS	6.181	(3.643, 10.487)	<0.001^a^
Initial shockable rhythm			
No			
Yes	1.875	(1.157, 3.039)	0.011^a^

CI indicates confidence interval; OR, adjusted odds ratio.

a *P*<0.05.

## Discussion

Our major findings are: (1) a 73% increase in the overall survival rate for OHCA in Detroit between 2014 and 2016; and (2) better OHCA survival if patients were female, if the arrest occurred in a public place, if the arrest was witnessed by EMS or bystanders, or if an initial shockable rhythm was present.

Two explanations can be provided for the significant improvement in OHCA survival rate and survival odds ratios (Table 4) during the study period. First, improvements in the chain of survival may have increased early CPR and AED application. Our data show a notable decrease in on‐scene time (Table 1), and increased CPR initiation and AED application by MFR (Table 2). These findings offer quantified improvements that reflect the impact of standardized on‐scene cardiac arrest protocols, an improved dispatch system and MFR training. These reforms likely led to a more rapidly initiated chain of survival that would have contributed to our documented increase in the percentage of defibrillated patients (Table 2) and earlier CPR. This may have contributed to our overall improved bystander‐witnessed survival, survival to hospital admission, and overall survival, as documented in other studies.^17^, ^18^, ^19^

Second, participating in the CARES registry not only provided data, but also kept all providers focused on cardiac arrest care improvement.^20^, ^21^ Indeed, the commitment of all stakeholders to improving Detroit cardiac arrest care was unprecedented, and every available resource was aligned toward achieving an increase in OHCA survival. Still, there is room for progress as Detroit's improved survival rates remain below the national average.^22^

For Detroit OHCA cases, survival rate was positively predicted by the arrest being witnessed, if the patient was female, if an initial shockable rhythm was present, and if the arrest occurred in a public place. Patients whose arrest was witnessed by an EMS responder were over 6 times more likely to survive than those whose arrests were unwitnessed. Patients whose arrests were witnessed by a bystander were >2.7 times more likely to survive. This is consistent with other studies, and these differences may be because of quicker dispatch and earlier OHCA interventions, stemming from the aforementioned changes to the Detroit EMS system.^23^, ^24^

Consistent with other studies, the location of arrest in Detroit is significantly associated with OHCA survival.^25^, ^26^ Among the 2359 OHCA cases we analyzed, 89.4% occurred in residential locations and experienced lower overall survival compared with OHCA in a public setting (also consistent with other studies).^27^ In Detroit, patients who arrested in a public setting were over 2 times more likely to survive than those who arrested in other settings, which may be because of less downtime, earlier EMS activation, and bystander use of life‐saving CPR and AED application.

Past research shows that the impact of location upon OHCA survival may be mediated by racial segregation. One recent study by Starks et al found that OHCA patients living in majority‐black neighborhoods had lower survival rates to hospital discharge.^28^ Residential segregation between blacks and other groups is extremely high in Detroit, and this segregation is associated with racial health disparities and high black mortality.^29^, ^30^, ^31^, ^32^ However, our calculated survival rates did not indicate a statistically significant difference in OHCA survival for blacks compared with other groups. This contrasts with other studies and may be related to our relatively small sample size, our single‐city analysis, lack of geographic analysis, and limited availability of clinical and social variables.^28^, ^33^ In the future, our ongoing data collection will allow us a thorough exploration of this issue.

There are several potential limitations to our study. First, our data are derived from CARES, which does not collect some clinical variables related to OHCA survival such as coronary risk factors, family history, medical therapy and adherence, comorbidity, and others. CARES, as a retrospective tool, may also miss some cases because of inadequacies or incompleteness of EMS records. Second, 84% of Detroit's population is black and in our data sample, 62.9% of 2359 OHCA patients were black.^34^ This demographic reality limits the applicability of our findings to other communities but provides a valuable point of comparison for similar urban settings in the greater United States.

## Conclusions

This study shows a dramatic increase in OHCA survival in Detroit between 2014 and 2016, and strongly suggests that EMS improvement and a more rapidly initiated chain of survival is critical to achieve such an outcome in urban settings with limited resources. It also provides a robust example of how better OHCA data collection and analysis can encourage a city‐wide effort to improve survival rates, while also contributing to the existing evidence base on the association of female sex, a public place, presence of an initial shockable rhythm, and presence of an EMS or bystander witness with OHCA survival. Future studies should measure how OHCA survival can be further improved by advanced prehospital interventions and elucidate the extent to which non‐clinical factors can be addressed to improve OHCA survival.

## Disclosures

None.
